# The Utilization of Complementary and Alternative Medicine among Saudi Older Adults: A Population-Based Study

**DOI:** 10.1155/2020/4357194

**Published:** 2020-08-07

**Authors:** Mohammad H. Aljawadi, Abdullah T. Khoja, Azzam D. AlOtaibi, Khalid Turki Alharbi, Muhannad Abdulwahed Alodayni, Mansour S. AlMetwazi, Azher Arafah, Sulaiman A. Al-Shammari, Tawfik A. Khoja

**Affiliations:** ^1^Department of Clinical Pharmacy, College of Pharmacy, King Saud University, Riyadh, Saudi Arabia; ^2^Public Health and Family Medicine Departments, College of Medicine, Al-Imam Muhammad Ibn Saud Islamic University (IMSIU), Riyadh, Saudi Arabia; ^3^Department of Medicine, College of Medicine, Al-Imam Muhammad Ibn Saud Islamic University (IMSIU), Riyadh, Saudi Arabia; ^4^Pharmacy Services, Prince Sultan Military Medical City, Riyadh, Saudi Arabia; ^5^College of Medicine, Imam Muhammad Ibn Saud Islamic University, Riyadh, Saudi Arabia; ^6^Department of Family and Community Medicine, College of Medicine, King Saud University, Riyadh, Saudi Arabia; ^7^Health Ministers' Council for the Cooperation Council States, Riyadh, Saudi Arabia

## Abstract

Background. Complementary and alternative medicine (CAM) is an integral part of patients' therapeutic experience worldwide. Among Saudi older adults, less is known about CAM utilization. Objectives. To determine the prevalence, patterns, and factors associated with CAM utilization among SOA. Methods. In the Saudi National Survey for Elderly Health (SNSEH), subjects were asked about CAM use during the last twelve months before the interview. CAM use was defined as any use of herbal products, acupuncture, bloodletting, cauterization, medical massage, bones manual manipulation, honey, or religious rituals. Demographic characteristics included gender, age, marital status, region, educational level, and residence area. In addition, multiple comorbidities were included as possible factors that may be associated with CAM use. Multivariable logistic regression was used to explore factors associated with CAM utilization. All statistical analyses were done using STATA v.14. Results. Out of 2946 respondents, 50.4% were males, the mean age was 70.3 ± 8.3 years, and 70% were illiterate. CAM use was prevalent (62.5%). The most common CAM types were herbal products (25.4%), acupuncture (21.2%), bloodletting (12%), honey (9.5%), cauterization (7.4%), medical massage and bones manual manipulation (4%), and traditional bone setting (2.1%). In the multivariable regression, age, gender, and marital status did not have an impact on the odds of using CAM. Subjects from rural areas were 2.92 times more likely to use CAM compared with subjects in urban areas (OR = 2.92; 95%CI: 2.28‐3.75). Subjects with metabolic disorders (OR = 0.50; 95% CI: 0.42‐0.60) or kidney disease were less likely to use CAM (OR = 0.30; 95%CI: 0.14‐0.64). About pain, CAM is used more in neck pain (OR = 1.69; 95%CI: 1.30‐2.21) and also used in back pain (OR =  1.22; 95%CI: 1.03‐1.46). Conclusions. CAM use was very prevalent among SOA. Clinicians and pharmacists must ask about CAM use among older adults as many of CAM may interact with patients medications.

## 1. Introduction

Complementary and alternative medicine (CAM) is a diverse group of interventions, practices, and products that are not considered as part of the usual care [1, 2]. They include different categories, such as herbal products, acupuncture, bloodletting, cauterization, medical massage, and manual manipulation of bones, as well as religious rituals and practices. The institute of medicine has listed more than one hundred interventions/practices under CAM [3]. While few interventions and practices, such as chiropractic, bloodletting, and massage therapy, have been regulated, many are being practiced without any form of regulations.

Despite the lack of regulations, the utilization of CAM is widespread worldwide. In Saudi Arabia, the prevalence of CAM use among adults ranged between 65% and 80% [4, 5]. This noticeable popularity of CAM has also been documented in other countries. For instance, Eisenberg et al. found that 34% of the US population have used CAM during a year [1]. Another study in the USA suggested that the prevalence of CAM utilization among adults can be as high as 62% [6]. Furthermore, 49% of the population in France, 46% in Germany, and 69% in Australia had used CAM treatment at some point in their lives [7, 8].

Certain factors have been associated with CAM use among older adults, including the availability of health caregivers in the area of residence, gender, education level, and ethnicity [9–11]. In addition, certain disease conditions such as insomnia, back pain, colds, and Alzheimer's disease were associated with the use of CAM among older adults [9, 11].

There are multiple reasons for CAM utilization, such as the assumption of CAM safety compared to conventional medicines, the perceived possible beneficial effects, having multiple medical conditions, and decreased functional activity [12–15]. Since many older adults have multiple medical conditions and decreased functional activities, one may expect high CAM utilization among this segment of the population. Many studies have documented patients have been using CAM for many conditions such as diabetes mellitus (DM), musculoskeletal, cardiac, neurological, urogenital diseases, and mood disorders [6, 16–18]. Therefore, with a high prevalence of use and multiple disease conditions, CAM users may be at risk of potentially harmful interactions between their conventional treatments and CAM products [1, 8, 14, 16, 19, 20].

For various reasons, older adults appear to be more inclined to use CAM compared to younger age groups. This propensity has been examined in various research worldwide. The higher prevalence of multiple medical conditions, decreased functional activity, the assumption of CAM safety compared to conventional medicines, perceived therapeutic effects are among the top reasons why CAM utilization appears to be more prevalent in older adults [12–15].

The Saudi geriatric population was estimated to be 3.2% of the total Saudi population in 2018 [21]. Therefore, exploring and understanding the extent and pattern of CAM utilization in Saudi Arabia is essential for healthcare providers and decision-makers to evaluate the current healthcare practices and to develop strategies to improve health outcomes and health service planning. Furthermore, there are very few published studies concerning CAM use among older adults in the middle east and none in Saudi Arabia.

Despite the aforementioned reasons, most studies focused on CAM utilization among adults or pediatric populations with no particular focus on CAM use in older adults. In Saudi Arabia, many studies looked at CAM use among samples where Saudi older adults (SOA) were only a subgroup of study samples rather than being the main focus of the study [18, 22–37]. For instance, Al-Faris and colleagues conducted a cross-sectional study that examined CAM use in 1408 Saudi adults in the Riyadh region. They found that 68% of the subjects had used CAM during the previous year and that the most critical determinant of CAM use was the perceived failure of conventional medical treatments [4]. Another study was conducted in 2010 in Riyadh city, and surrounding governorates found that among 518 participants, 85% of subjects or one of their family members has used some form of CAM before [5]. However, these studies represent a sample of SOA and cannot be representative of the whole SOA as many of them were not nationally representative samples. Besides, none of those above studies were able to look at the relationship between CAM use and overall mortality despite the fact that few studies have documented increase perception of death with CAM or death due to CAM utilization. In contrast, others have shown survival benefits due to the introduction of CAM in patients' therapeutic plan [38–41]. Therefore, the objectives of this study are to determine the prevalence and factors associated with CAM utilization among SOA. In addition, to determine the effect of CAM utilization on mortality among SOA. The outcomes of this study are essential for healthcare providers and decision-makers to evaluate healthcare practices and to develop strategies that improve health outcomes among SOA.

## 2. Methods

### 2.1. Data Source

Data was based on the Saudi National Survey for Elderly Health (SNSEH). It is a nationwide, representative, population-based, cross-sectional survey of SOA who are 60 years of age or older. The survey was conducted between 2006 and 2007 and aimed to evaluate the health status and healthcare provided to SOA. The survey is one of the most extensive national health surveys designed to capture health-related information among older adults in Saudi Arabia. The survey was described in detail in an earlier study [42].

### 2.2. Exposure

In this study, CAM use was defined as any use of herbal products, acupuncture, bloodletting, cauterization, medical massage, manual manipulation of bones, honey, or religious rituals during the previous twelve months of the interview. Since religious rituals are very common in the Saudi Arabian society, they were defined as going to a scholar for performing the prayers and not self-prayers. The categories of CAM were based on the cultural practices in Saudi Arabia. The subjects were asked if they have been using any of these categories as a CAM.

### 2.3. Potential Factors Associated with CAM Use

Demographic characteristics included gender, age, marital status, region, educational level, monthly income, and the area of residence. Besides, DM, depression, hypertension, cognitive impairment, obesity, cancer, metabolic disorders, acute and chronic kidney disease, and neck and back pain were also included as possible factors that may be associated with CAM use.

### 2.4. Study Design

This was a cross-sectional study. The sample included Saudi citizens of sixty years of age or older. To have a nationally representative sample, a complex survey approach was used where the point estimate of disease or disability was assumed to be 10%, 95% level of significance, precision degree 0.02, design effect of 2.5, and 80% response rate 80%. Consequently, the suggested sample size was 2704 subjects. A cluster size of 31 subjects was chosen, considering an interclass correlation of 0.05. Furthermore, through the application of Probability Proportional to Size (PPS) sampling and systematic random sampling, 88 clusters were chosen. The cluster frame was created by using a catchment area of primary healthcare centers. Within the cluster frame, a cluster map was used to select both random streets and random household. Trained interviewers contacted the picked-out individuals for two visits to carry on the interview and the physical examinations. Subjects were asked to sign an informed consent if they agreed to participate. However, proxies, that is, legal guardians, were asked to sign in the informed consent form in case of subjects with cognitive impairments. In addition, “proxies were mainly used when physical or intellectual impairments prevented the subject from directly answering the interviewer. Nevertheless, proxies were not used if the questionnaire assists the mental or physical patient status, such as depression scale and cognitive abilities.” Lastly, standardized survey weights based on census data were calculated to account for oversampling, nonresponse, and postsampling stratification.

### 2.5. Statistical Analyses

The cohort was described through frequencies and percentages for all categorical variables. All percentages were adjusted by standardized survey weight to incorporate nonresponse, oversampling, and postsampling stratification. The Chi-square test was used to test the association between the variables of interest and CAM. Bivariable and multivariable logistic regressions were used to explore factors associated with CAM utilization among SOA. Both C-statistic and Hosmer–Lemeshow tests were used to assess the goodness of fit of the logistic regression model.

#### 2.5.1. Survival Analysis

Subjects were followed up until mid-2015 to determine their vital status and time to death. Cox proportional hazard was used to assess the effect of CAM use on nine-year survival adjusting for age, gender, diabetes, hypertension stroke, obesity, cancer, ischemic heart diseases, cognitive impairment, renal failure, place of residence, smoking status, and depression. Subjects were censored if they did not die at the end of the follow-up period. Both Groennesby and Borgan test and graphical plotting of Nelson–Aalen cumulative hazard against Cox-Snell Residuals were used to determine the model's goodness of fit [43–45]. All statistical analyses were done using (STATA 14) [46]. The institutional review board approved the study at Imam Mohammad Bin Saud Islamic University (HAPO-01-R-011).

## 3. Results

The demographic characteristics of the subjects are shown in Table 1. The overall sample included 2,946 individuals from the thirteen administrative regions of Saudi Arabia. Out of 2946 respondents, 1759 (50.4%) were males, the mean age was 70.3 ± 8.3 years, and 70% were illiterate.

**Figure 1:**
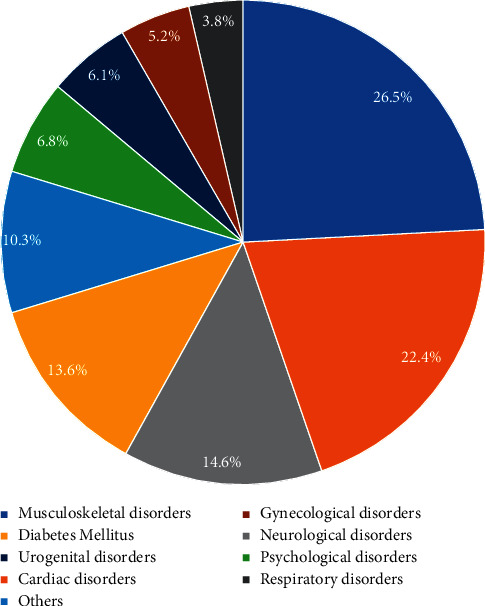
The most common disorders for using complementary and alternative medicines among Saudi older adults (*N* = 2,946).

During the twelve months before the interview, the overall prevalence of CAM use was 62.5%. There was no noticeable difference in CAM use between men (63.3%) and women (62.5%) (*P*=0.627). The most common CAM types were herbal products (25.4%), acupuncture (21.2%), bloodletting (12%), honey (9.5%), cauterization (7.4%), medical massage and manual manipulation of bones (4%), and lastly traditional bone setting (2.1%). These groups were not mutually exclusive as subjects may have used more than one CAM type.  Figure 1 shows the most common medical conditions reported among CAM users.

**Figure 2:**
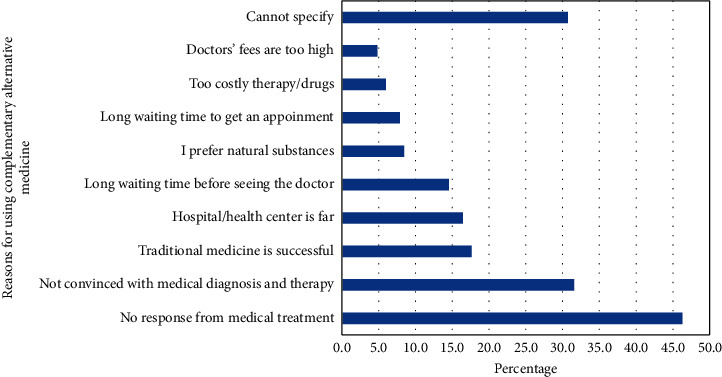
The most common reasons for using complementary and alternative medicine over conventional treatment among Saudi older adults (*N* = 2,946).

Among users of CAM, the most common medical conditions reported were musculoskeletal disorders (26.5%), followed by cardiac problems (22.4%), neurological problems (14.6%), and diabetes mellitus (13.6%), as shown in Figure 1. On the other hand, the least common reason to seek CAM was respiratory problems (4%). Furthermore, Figure 2 depicts the common reasons for choosing CAM over conventional medical treatments.

In the multivariable regression, age and gender did not have an impact on the odds of using CAM. Compared to subjects who did not smoke, the odds of CAM use were higher among ex-smokers (OR = 1.43; 95% CI: 1.09–1.88), as well as daily smokers (OR = 1.71; 95% CI: 1.14–2.55) but not among occasional smokers (OR = 1.20; 95% CI: 0.47–3.04). Subjects from rural areas were 2.57 times more likely to use CAM compared to subjects in urban areas (OR = 2.57; 95% CI: 1.98–3.33).

Regarding regional variations in SA, SOA in the western and eastern regions were found to be frequent CAM users compared to the central area (OR = 1.68; 95% CI: 1.32–2.13; OR = 2.18; 95% CI: 1.62–2.95, resp.) (Table 2). While the southern part is the least in CAM using (OR = 0.59; 95% CI: 0.46–0.76).

Subjects with metabolic disorders (OR = 0.51; 95% CI: 0.42–0.61) or kidney disease were less likely to use CAM (OR = 0.30; 95% CI: 0.14–0.64). In the case of pain management, CAM is used more in neck pain (OR = 1.69; 95% CI: 1.30–2.21) and also used in back pain (OR = 1.26; 95%: 1.05–1.50). Cognitive impairment was associated with lower odds of CAM utilization among SOA (Table 2). Lastly, the C-statistic for the multivariable regression was 74.7%, and the Hosmer–Lemeshow test was not significant (*P*=0.07).

**Figure 3:**
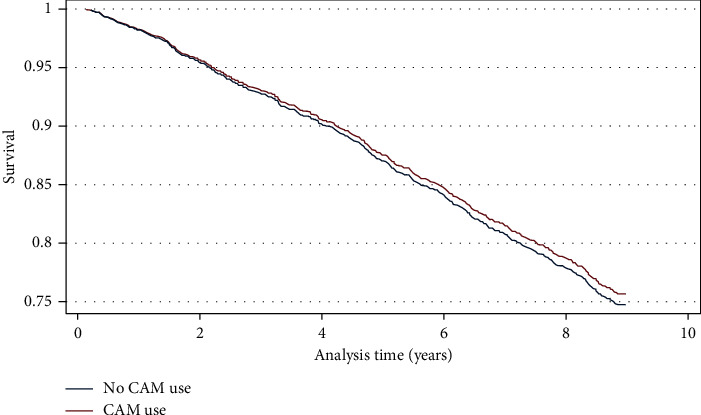
Survival curves based on Cox proportional hazard regression for the effect of CAM use on survival among Saudi older adults between 2006 and 2015.

**Figure 4:**
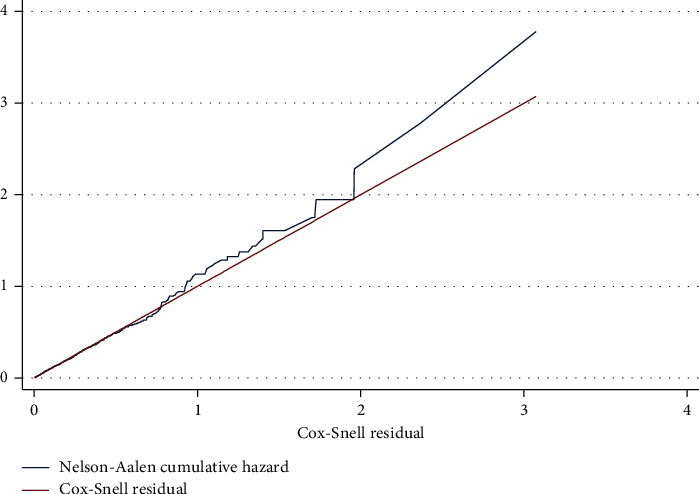
Goodness of fit test by plotting of Nelson–Aalen cumulative hazard against Cox-Snell Residuals.

With regard to nine-year survival, 2,047 subjects had their vital status available for analysis. There was no statistical difference in mortality between subjects who used CAM (29.79%) and more prominently those who did not (29.77%; *P*=0.992). In the multivariable Cox proportional hazard regression, the hazard ratio of death between 2006 and 2015 was 0.96, with a 95%CI (0.81–1.14) indicating no difference in mortality between the two groups (Figure 3). The graphical plotting of Nelson–Aalen cumulative hazard against Cox-Snell Residuals (Figure 4) shows a good fit of the model by the superimposition of the two plots over each other. In addition, the Groennesby and Borgan test that was based on ten quantiles of risk was insignificant (*P*=0.5274), indicating a good fit of the model.

## 4. Discussion

In this study, CAM utilization was prevalent among Saudi older adults at 62.5%. The prevalence of CAM use varies between studies and countries. For example, In the USA, Clarke et al. reported that 40% to 60% of US citizens had used CAM during the twelve months before the administration of the national health interview surveys [47]. In Europe, CAM utilization over one year was 23% in Denmark, 49% in France, and 46% in Germany [7]. Such differences in the prevalence can be related to demographics [48], locality of herbs, or the difference CAM definition. For instance, bloodletting, as a CAM in Saudi Arabia, constituted around 19% of all CAM used by SOA. On the other hand, none of those above countries considered bloodletting as a CAM. Such a technique started to become noticed again only after Olympics athletes began to use dry and wet cupping during the 2016 Olympics games despite weak scientific evidence supporting its use in sports [49].

Both the C-statistic (74.7%) and the Hosmer–Lemeshow test (*P*=0.07) indicated a good fit of the multivariable logistic regression. The regression model revealed that CAM utilization was not significantly different by sex. This finding is consistent with many of the published works in the literature [1, 12, 16, 20, 50]. However, many studies have found that CAM use was more prevalent in young females due to mass media affection, family members, and friends [5, 8, 51–53].

The facts that 46.3% of subjects stated that the reason for taking CAM is having no response to medical treatment and 31.6% were not convinced with the medical diagnosis and therapy necessitate the importance of strengthening the patient-physician relationship in order to discuss such perceptions and beliefs. Strong patient-physician relationship has shown to improve patient understanding of their disease, medications' role in improving their health, and their quality of life [54–56].

Furthermore, the aforementioned patients' beliefs about having no response to medical treatment (46.3%) or not being convinced with the medical diagnosis or therapy (31.6%) open the opportunity for collaborative services between physicians and pharmacists through medication therapy management clinics. These clinics can address patients' concerns regarding their current medications to improve their medication adherence over unsupported CAM use. Pharmacist-physician collaborative practices have been successful in improving outcomes among patients with chronic diseases such as those found among older adults [57–61]. Therefore, the pharmacist-physician collaboration can create a safety net for patients to express their concerns regarding their treatment plan, which in turn can reduce unnecessary use of CAM, increase adherence to treatment, and solicit a change in treatment plan when necessary.

In this study, the top four types of CAM were herbal products, acupuncture, bloodletting, and religious/Quranic rituals (Figure 1). Using a random sample of Riyadh residents, Elolemy et al. reported that herbal products (59%), prayers (54.6%), honey products (54.3)%, and bloodletting (35.7%) occupied the top four CAM in [5]. The difference between this study and the study above may be explained not only by the difference between Riyadh region and the whole kingdom but also by the subjects' case-mix as many participants were younger than this study's subjects.

Furthermore, an explanation for the high prevalence of acupuncture among SOA could be the subjects' understanding of acupuncture itself. In more detail, the literal translation of acupuncture in the Arabic language is “Chinese Injections.” With 70% illiteracy among subjects, it is most likely that the participants understood “Chinese Injections” as one form of injections that they may have been exposed to in the hospital leading to the inflated prevalence.

In the United States, during 2007, the most common types of CAM utilized by the US residents were natural products (17.7%) and deep breathing exercises (12.7%) [2]. The results of this study, together with the studies above, indicate the cruciality of asking patients about the use of herbal products to avoid any therapy failure due to drug-herb interactions, which have been frequently documented in the literature [62–73]. Moreover, with almost 18% of subjects using CAM because of their beliefs about its usefulness (Figure 4) which may not be substantiated by scientific evidence, patients may abandon their prescribed medications and continue using CAM, leading to worsening of their disease status. For instance, Huiart and colleagues found that, among 233 patients with cancer who were receiving aromatase inhibitors (AIs), the hazard ratio of discontinuing AIs was 3.2 times greater among subjects using CAM compared to subjects who did not (95% CI: 1.5–6.9). In their conclusion, the investigators stated that “some patients may use CAM not as a complementary treatment, but as an alternative to conventional medicine.” [74]. Nevertheless, few patients share their CAM use with physicians. For instance, Sullivan et al. have reported that only 33% of patients have told their physicians about their CAM use. The main reason for nondisclosure was “doctor never asked” [75]. Furthermore, few studies have reported that, among patients with cancer who used CAM as the sole therapy for their cancer, the risk of death was higher than patients who did not [76–78].

In this study, daily smoking was associated with a 71% increase in the odds of CAM utilization (OR = 1.71; 95 CI%: 1.15–2.5). Multiple reasons may explain such behavior; for instance, Sood et al. reported that 27% of smokers have reported using CAM for smoking cessation [79, 80]. Acupuncture has been shown to reduce daily cigarette use and increased the success of quitting [81]. Moreover, since smoking increases mucus accumulation in the lungs, many smokers may end up using herbal products as expectorants or mucolytics [82, 83].

Regardless of CAM type, subjects who were living in rural areas were 2.57 times more likely to use CAM than the subjects in urban areas (OR = 2.57; 95% CI: 1.98–3.33). The utilization of CAM in rural areas has been documented in different countries, including Malaysia, Australia, Nigeria, Japan, and the USA [84–88]. While almost 17% of subjects reported that the reason for CAM use is the remoteness of the hospital or health center, the regression results showed that living in rural areas was associated with increased CAM use even after controlling for healthcare accessibility to facilities such as pharmacies, healthcare centers, or hospitals. Therefore, it is most likely that among SOA, cultural beliefs and family traditions that may have been thawed in urban cities are a more prominent influencer of CAM use in rural areas than access to care. Consequently, future research should explore what beliefs and cultural habits influence the utilization of CAM among older adults in Saudi Arabia.

Concerning regional variations and the utilization of CAM (Table 2), this is the first study documenting regional differences in CAM use among SOA. Subjects living in the western and eastern regions were more likely to utilize CAM than subjects in the central region. It is possible that the closeness of people living in the eastern region to other Gulf countries has exposed them to CAM formulas and uses more than those in the central region. Similarly, the exposure of subjects in the western area to people coming for Hajj from almost all countries of the world may have influenced their utilization pattern. The pattern of regional variation has been reported in other countries. For instance, Nguyen and colleagues have reported that CAM utilization was higher among people living in the Central Appalachia region than the national average in the USA [89]. However, Williams et al. argued that geography itself does not explain differences in utilization, but factors such as demographic characteristics and unmet healthcare need perceived by the individuals play a more significant role [48]. Therefore, more place-specific research is needed before the hypotheses mentioned above are confirmed.

Despite the plethora of scientific research exploring CAM use among patients with cognitive impairment, there is a scarcity in such research that examines whether cognitive impairment increases or decreases CAM utilization. Almost all studies that studied cognitive impairment and CAM utilization focused on what is being used and its efficacy among patients with cognitive impairment [90–96]. We found that subjects with any level of cognitive impairment were less likely to utilize CAM than healthy subjects (Tables 1 and 2). However, it is noteworthy that almost around 50% of patients with cognitive impairments have used CAM (Table 1).

A similar trend was observed with disorders that required a strict diet. Patients with these disorders were less likely to utilize CAM (OR = 0.51; 95% CI: 0.42–0.61; acute or chronic kidney injuries: OR = 0.30; 95% CI: 0.14–0.64)). In addition, patients with cancer were less likely to use CAM compared to subjects without cancer (OR = 0.40; 95% CI: 0.22–0.72). However, almost 50% of subjects on metabolic disorders, 40% of subjects with kidney injuries, and 45% of patients with cancer have utilized CAM (Table 1). Patients with the abovementioned conditions could be more cautious in using CAM due to immediate effects on their health.

After adjusting for age, gender, diabetes, hypertension, stroke, obesity, cancer, ischemic heart diseases, cognitive impairment, renal failure, place of residence, smoking status, and depression, the use of CAM had neither any survival advantage nor any increase in mortality among SOA. Almost no study has demonstrated a survival benefit with CAM utilization alone while few studies have reported that a combination of CAM with conventional therapy, that is, integrated therapy, has shown survival benefits among patients with polymyositis, dermatomyositis, and advanced ovarian cancer [38, 39]. On the other hand, few studies have reported that CAM use has led to a higher refusal of conventional therapies in cancer, leading to an increase in mortality [76–78].

Since Groennesby and Borgan test and the plot of Nelson–Aalen cumulative hazard against Cox-Snell Residuals (Figure 4) indicated a good fit of the regression model, future studies should examine the effect of CAM use on the activity of daily livings as well as the quality of life rather than survival.

The high prevalence of CAM utilization among SOA mandates the implementation of the following recommendations: first, it is essential to remind the patients and their caregivers about disclosing any CAM use to their pharmacists or physicians. Although this study showed neither beneficial nor harmful effect of CAM use on survival, such disclosure may improve patient care by avoiding the use of products with unsubstantiated evidence, decreasing unnecessary out-of-pocket cost, understanding patients' perception and believes that led to CAM use or, on the other hand, exploring potential additive or synergistic effect for the integrated therapy. Second, future studies should focus on exploring the impact of each CAM on the activities of daily living and quality of life. Third, with a high prevalence, it is vital to regulate the market of CAM in Saudi Arabia to reduce counterfeiting and ensure the authenticity of marketed products. Lastly, a collaboration between the national center of complementary medicine, the universities, and the National & Gulf Center for Evidence-Based Health Practice should determine priorities of CAM-related research and support funding of this area of research.

Certain limitations should be taken into consideration while interpreting these results. First, due to the cross-sectional nature of the study, causality cannot be tested. Second, the lack of more details about the nature of herbal products used limits the ability to determine the most common possible herbal-drug interactions among SOA. Third, since the data was collected in 2007, the pattern of CAM utilization may have changed. Nonetheless, this study still provides valuable baseline information about CAM use among SOA. Such a baseline is essential to determine the trend of use over time.

In conclusion, this study is the first nationally representative sample that explored CAM utilization among SOA. The use of CAM was very prevalent among SOA, especially in rural areas. Despite this, the use of CAM did not demonstrate any survival benefit over nine years. Future research should focus on replicating the study to identify changes in patterns of utilization over time; exploring factors associated with the observed geographic variations will be very beneficial in directing interventions and policy decisions across the regions and determining whether or not CAM use has an impact on the quality of life rather than survival.

## Figures and Tables

**Table 1:** Univariable and bivariable analyses of complementary and alternative medicine utilization among Saudi older adults (*N* = 2,946).

Subjects characteristics	Total *N* = 2,946		No CAM use *n* = 1096 (37.5%)		CAM use *n* = 1850 (62.5%)		*χ* ^2^, *P* value
	*n*	**(%)**	*n*	**(%)**	*n*	**(%)**	

Gender							**0.627**
Female	1187	**(49.6)**	443	**(38.3)**	744	**(61.7)**	
Male	1759	**(50.4)**	653	**(36.7)**	1106	**(63.3)**	
Age (years)							**0.918**
60–65	1072	**(37.7)**	421	**(38.6)**	651	**(61.4)**	
66–70	697	**(23.2)**	250	**(36.7)**	447	**(63.3)**	
71–75	504	**(16.6)**	185	**(37.1)**	319	**(62.9)**	
76–80	342	**(11.3)**	118	**(36.2)**	224	**(63.8)**	
81–85	181	**(6.1)**	67	**(36.8)**	114	**(63.2)**	
86–90	101	**(3.4)**	33	**(33.8)**	68	**(66.2)**	
>90	49	**(1.8)**	22	**(43.6)**	27	**(56.4)**	
Education level							**0.212**
Illiterate	1943	**(69.6)**	718	**(38)**	1225	**(62)**	
Less than 8 years	705	**(21.8)**	247	**(34)**	458	**(66)**	
Intermediate or high	222	**(6.5)**	102	**(44.4)**	120	**(55.6)**	
University or higher	76	**(2.1)**	29	**(34.5)**	47	**(65.5)**	
Smoking history							**0.006**
Never smoke	2375	**(82.9)**	932	**(39.3)**	1443	**(60.7)**	
Ex-smoker	385	**(11.4)**	113	**(29.5)**	272	**(70.5)**	
Occasional smoker	22	**(0.7)**	8	**(30.7)**	14	**(69.3)**	
Daily smoker	164	**(5)**	43	**(26.2)**	121	**(73.8)**	
Income (SR)							**0.859**
>10000	181	**(5.6)**	82	**(41.5)**	99	**(58.5)**	
9,999–7,500	140	**(4.2)**	52	**(35.4)**	88	**(64.6)**	
7,499–5,000	318	**(9.6)**	114	**(35.5)**	204	**(64.5)**	
4,999–2,500	752	**(24.2)**	269	**(36.5)**	483	**(63.5)**	
<2,500	1555	**(56.5)**	579	**(38)**	976	**(62)**	
Marital status							**0.193**
Monogamy	1741	**(57.2)**	641	**(36.8)**	1100	**(63.2)**	
Polygamy	430	**(13.2)**	174	**(40.5)**	256	**(59.5)**	
Widowed	584	**(23.1)**	226	**(39.8)**	358	**(60.2)**	
Single	115	**(3.6)**	38	**(33.2)**	77	**(66.8)**	
Separated	76	**(2.9)**	17	**(24.2)**	59	**(75.8)**	
Residence							**0.132**
Urban	2398	**(80.1)**	954	**(40.3)**	1444	**(59.7)**	
Rural	548	**(19.9)**	142	**(26.3)**	406	**(73.7)**	
Regions							**0.390**
Central	753	**(23.2)**	308	**(41.2)**	445	**(58.8)**	
Western	874	**(31.1)**	257	**(28.8)**	617	**(71.2)**	
Eastern	393	**(13.7)**	111	**(34.8)**	282	**(65.2)**	
Southern	731	**(25.1)**	314	**(42)**	417	**(58)**	
Northern	195	**(6.9)**	106	**(53.2)**	89	**(46.8)**	
BMI WHO categories							**0.990**
Less than 18.5	50	**(1.8)**	18	**(36.2)**	32	**(63.8)**	
From 18.5 to 25	872	**(28.6)**	319	**(37.3)**	553	**(62.7)**	
From 25 to 30	1132	**(37.5)**	431	**(37.9)**	701	**(62.1)**	
More than 30	892	**(32.1)**	328	**(37.2)**	564	**(62.8)**	
Cognitive impairment^ℵ^							**0.022**
Normal cognition	2474	**(82)**	872	**(35.2)**	1602	**(64.8)**	
Mild	312	**(11.9)**	144	**(47.7)**	168	**(52.3)**	
Moderate	103	**(4)**	49	**(46.3)**	54	**(53.7)**	
Severe	57	**(2.2)**	31	**(52.5)**	26	**(47.5)**	
Depression^**◊**^							**0.146**
Normal (<5)	2192	**(73.3)**	853	**(39.2)**	1339	**(60.8)**	
Suggestive (5–10)	682	**(24.2)**	218	**(32.4)**	464	**(67.6)**	
Depression (>10)	72	**(2.6)**	25	**(37.5)**	47	**(62.5)**	
Diabetes mellitus							**0.905**
No	1625	**(55.6)**	597	**(37.3)**	1028	**(62.7)**	
Yes	1321	**(44.4)**	499	**(37.7)**	822	**(62.3)**	
Hypertension							**0.519**
No	1072	**(35.5)**	416	**(38.6)**	656	**(61.4)**	
Yes	1874	**(64.5)**	680	**(36.9)**	1194	**(63.1)**	
Stroke							**0.226**
No	2906	**(98.7)**	1077	**(37.3)**	1829	**(62.7)**	
Yes	40	**(1.3)**	19	**(48.9)**	21	**(51.1)**	
Cancer							**0.048**
No	2892	**(98.1)**	1067	**(37.2)**	1825	**(62.8)**	
Yes	54	**(1.9)**	29	**(54.3)**	25	**(45.7)**	
Ischemic heart disease							**0.589**
No	2838	**(96.5)**	1053	**(37.4)**	1785	**(62.6)**	
Yes	108	**(3.5)**	43	**(40.6)**	65	**(59.4)**	
Metabolic disorders							**< 0.001**
No	2106	**(71.3)**	685	**(32.1)**	1421	**(67.9)**	
Yes	840	**(28.7)**	411	**(50.8)**	429	**(49.2)**	
Acute kidney failure and chronic kidney disease							**0.012**
No	2912	**(98.9)**	1076	**(37.2)**	1836	**(62.8)**	
Yes	34	**(1.1)**	20	**(61)**	14	**(39)**	
Neck pain							0.016
No	2539	**(85.4)**	990	**(39.2)**	1549	**(60.8)**	
Yes	407	**(14.6)**	106	**(27.6)**	301	**(72.4)**	
Back pain							**0.024**
No	1694	**(56.3)**	685	**(41)**	1009	**(59)**	
Yes	1252	**(43.7)**	411	**(33)**	841	**(67)**	
Blood in stools							**0.091**
No	2882	**(97.9)**	1065	**(37.2)**	1817	**(62.8)**	
Yes	64	**(2.1)**	31	**(49.8)**	33	**(50.2)**	
Headache							**0.315**
No	1153	**(37.4)**	469	**(39.9)**	684	**(60.1)**	
Yes	1793	**(62.6)**	627	**(36)**	1166	**(64)**	
Fainting							**< 0.001**
No	2490	**(84.1)**	1010	**(40.9)**	1480	**(59.1)**	
Yes	456	**(15.9)**	86	**(19.6)**	370	**(80.4)**	
Feeling fatigued							**0.022**
No	363	**(13.2)**	177	**(49.3)**	186	**(50.7)**	
Yes	2583	**(86.8)**	919	**(35.7)**	1664	**(64.3)**	
Availability and accessibility of a pharmacy within your locality or village?							**0.061**
Not available nor accessible	222	**(8)**	91	**(40.3)**	131	**(59.7)**	
Available only	568	**(20)**	166	**(29.4)**	402	**(70.6)**	
Accessible only	179	**(6.4)**	38	**(22.5)**	141	**(77.5)**	
Available and accessible	1977	**(65.7)**	801	**(41)**	1176	**(59)**	
Availability and accessibility of a health center within your locality or village?							**0.549**
Not available nor accessible	95	**(3.4)**	33	**(35.9)**	62	**(64.1)**	
Available only	434	**(15.4)**	136	**(31.6)**	298	**(68.4)**	
Accessible only	148	**(5.1)**	52	**(35.6)**	96	**(64.4)**	
Available and accessible	2269	**(76.1)**	875	**(38.9)**	1394	**(61.1)**	
Availability and accessibility of a hospital within your locality or village?							**0.689**
Not available nor accessible	203	**(7.1)**	63	**(27.8)**	140	**(72.2)**	
Available only	717	**(25.2)**	265	**(36.5)**	452	**(63.5)**	
Accessible only	240	**(8.2)**	85	**(35.9)**	155	**(64.1)**	
Available and accessible	1786	**(59.5)**	683	**(39.3)**	1103	**(60.7)**	

SR : Saudi riyals in 2007; BMI : body mass index; WHO : World Health Organization;^ℵ^ based on Short Portable Mental Status Questionnaire (SPMSQ); ◊ based on Geriatric Depression Scale; ^*∗*^*P* < 0.05, ^*∗∗*^*P* < 0.01, and ^*∗∗∗*^*P* < 0.001.

**Table 2:** Multivariable logistic regression of factors associated with utilization of complementary alternative medicine among Saudi older adults 2006–2007 (*N* = 2,946).

Characteristics	OR	95% CI
Male	0.919	[0.728,1.158]
Age (60–65)	1	[1,1]
66–70	1.051	[0.842,1.311]
71–75	1.064	[0.828,1.366]
76–80	1.18	[0.880,1.583]
81–85	0.964	[0.659,1.412]
86–90	1.302	[0.786,2.157]
>90	0.687	[0.354,1.330]
Level of education (ref : illiterate)	1	[1,1]
Less than 8 years	1.027	[0.819,1.289]
Intermediate to high school	0.606^*∗∗*^	[0.417,0.881]
University or higher	0.921	[0.503,1.689]
Smoking history (ref : never smoke)	1	[1,1]
Ex-smoker	1.430^*∗*^	[1.089,1.877]
Occasional smoker	1.195	[0.471,3.037]
Daily smoker	1.707^*∗∗*^	[1.144,2.547]
Income (SR) (ref: < 2,500)	1	[1,1]
>10000	1.018	[0.677,1.530]
9,999–7,500	1.536	[0.998,2.363]
7,499–5,000	1.222	[0.903,1.655]
4,999–2,500	1.165	[0.945,1.438]
Marital status (ref : monogamy)	1	[1,1]
Polygamy	0.826	[0.647,1.054]
Widowed	0.85	[0.661,1.093]
Single	0.915	[0.588,1.424]
Separated	2.399^*∗∗*^	[1.297,4.438]
Rural (ref : urban)	2.569^*∗∗∗*^	[1.981,3.330]
Five regions of Saudi Arabia (ref : central)	1	[1,1]
Western	1.678^*∗∗∗*^	[1.322,2.129]
Eastern	2.183^*∗∗∗*^	[1.615,2.952]
Southern	0.592^*∗∗∗*^	[0.459,0.763]
Northern	0.763	[0.535,1.087]
BMI WHO categories (Ref: 18.5 to 25)	1	[1,1]
< 18.5	1.397	[0.717,2.720]
From 25 to 30	1.276	[0.655,2.483]
More than 30	1.329	[0.678,2.604]
Cognitive impairment^ℵ^ (ref : normal)	1	[1,1]
Mild	0.563^*∗∗∗*^	[0.424,0.746]
Moderate	0.471^*∗∗*^	[0.291,0.759]
Severe	0.394^*∗∗*^	[0.211,0.734]
Depression (ref : normal)^◊^	1	
Suggestive of depression	1.261^*∗*^	[1.013,1.570]
Depression	1.519	[0.842,2.738]
Diabetes mellitus	0.882	[0.742,1.049]
Hypertension	1.087	[0.909,1.299]
Stroke	0.436^*∗*^	[0.213,0.893]
Cancer	0.395^*∗∗*^	[0.216,0.722]
Ischemic heart disease	0.845	[0.544,1.314]
Metabolic disorders	0.505^*∗∗∗*^	[0.422,0.606]
Acute kidney failure and chronic kidney disease	0.303^*∗∗*^	[0.143,0.644]
Neck pain	1.691^*∗∗∗*^	[1.292,2.212]
Back pain	1.257^*∗*^	[1.051,1.503]
Blood in stools	0.543^*∗*^	[0.312,0.945]
Headache	1.083	[0.906,1.294]
Feeling fainted	3.202^*∗∗∗*^	[2.424,4.228]
Feeling fatigued	1.725^*∗∗∗*^	[1.345,2.212]
Availability of pharmacy (ref: not available)	1	[1,1]
Available only	2.188^*∗∗*^	[1.330,3.600]
Accessible only	2.894^*∗∗∗*^	[1.638,5.111]
Available and accessible	0.936	[0.602,1.455]
Availability of healthcare center (ref: not available)	1	[1,1]
Available only	0.996	[0.526,1.886]
Accessible only	0.924	[0.436,1.960]
Available and accessible	0.994	[0.546,1.811]
Availability of hospital (ref: not available)	1	[1,1]
Available only	0.556^*∗*^	[0.338,0.914]
Accessible only	0.686	[0.388,1.213]
Available and accessible	1.096	[0.687,1.748]
Observations	2,946	

OR : odds ratio; 95% CI: 95% confidence interval; BMI : body mass index; WHO : World Health Organization; ^ℵ^ based on Short Portable Mental Status Questionnaire (SPMSQ); ◊ based on Geriatric Depression Scale, ^*∗*^*P* < 0.05, ^*∗∗*^*P* < 0.01, and ^*∗∗∗*^*P* < 0.001.

## Data Availability

The data used to support the findings of this study are available from the corresponding author upon request.
